# Active Management of Third Stage of Labour Saves Facility Costs in Guatemala and Zambia

**Published:** 2006-12

**Authors:** Judith T. Fullerton, Kevin D. Frick, Linda A. Fogarty, Joy D. Fishel, Donna M. Vivio

**Affiliations:** ^1^ Project Concern International, San Diego, CA 92123, USA; ^2^ Johns Hopkins Bloomberg School of Public Health, Baltimore, MD 21205, USA; ^3^ JHPIEGO, 1615 Thames Street, Baltimore, MD 21231-3492, USA; ^4^ ORC Macro, 11785 Beltsville Drive, Calverton, MD 20705, USA

**Keywords:** Postpartum haemorrhage, Labour stage, Third, Cost and cost analysis, Cost-benefit analysis, Guatemala, Zambia

## Abstract

This study calculated the net benefit of using active management of the third stage of labour (AMTSL) rather than expectant management of the third stage of labour (EMTSL) for mothers in Guatemala and Zambia. Probabilities of events were derived from opinions of experts, publicly available data, and published literature. Costs of clinical events were calculated based on national price lists, observation of resources used in AMTSL and EMTSL, and expert estimates of resources used in managing postpartum haemorrhage and its complications, including transfusion. A decision tree was used for modelling expected costs associated with AMTSL or EMTSL. The base case analysis suggested a positive net benefit from AMTSL, with a net cost-saving of US$ 18,000 in Guatemala (with 100 lives saved) and US$ 145,000 in Zambia (with 467 lives saved) for 100,000 births. Facilities have strong economic incentives to adopt AMTSL if uterotonics are available.

## INTRODUCTION

Active management of the third stage of labour (AMTSL) refers to a sequence of clinical actions taken by the skilled birth attendant to facilitate the delivery of the placenta, by promoting uterine contraction and placental expulsion. AMTSL, an alternative to physiological, or expectant management of the third stage of labour (EMTSL), reduces the incidence of postpartum haemorrhage which is the leading cause of maternal mortality worldwide (1, 2).

Non-governmental organizations (NGOs) have been working with ministries of Health and Education in Guatemala and Zambia for several years on strategies to improve maternal and child health. The U.S. Agency for International Development (USAID)-funded Maternal and Neonatal Health Global Programme, implemented by the JHPIEGO Corporation, USA, selected AMTSL as a priority intervention in these two countries. The maternal mortality ratio (MMR) reported in the most recent demographic and health survey (DHS) conducted in Guatemala in 1999 (3) is 190 per 100,000 livebirths, or 240 when adjusted for method of computation (4), and 153 per 100,000 livebirths in a recent reproductive age mortality study (5). In Zambia, the MMR is 729 per 100,000 livebirths (750 adjusted) (6).

The promotion of AMTSL has occurred through policy advocacy, pre-service, and continuing education of healthcare providers. Experience has demonstrated that skilled birth attendants can be readily taught the techniques of AMTSL. However, to date, no cost-bene-fit or cost-effectiveness analysis has been conducted to indicate whether the additional costs of personnel and material resources relating to the use of AMTSL would be offset by costs avoided by achieving better maternal outcomes. Averted costs result from avoiding postpartum haemorrhage and, in turn, conserving resources required for the management of postpartum haemorrhage. An analysis was undertaken to fill this information gap and to demonstrate the utility of the intervention in two countries that varied greatly in their geographic and demographic context. Evidence of the potential cost-savings of routine AMTSL for health facilities could help foster the wide adoption of this lifesaving technique.

## MATERIALS AND METHODS

### Definition of study variables

A set of three clinical interventions distinguishes AMTSL from expectant management. EMTSL, also known as conservative or physiological management, involves waiting for signs of separation of the placenta and allowing the placenta to deliver spontaneously. The early definition of AMTSL included: (a) intramuscular administration of a prophylactic uterotonic after the delivery of the baby; (b) early cord-clamping and cutting; and (c) controlled traction on the umbilical cord (7–9). Oxytocin was the synthetic agent used in the earliest clinical trials (10, 11). Other uterotonics (ergometrine, syntometrine, and misoprostol) (12–17) and alternative routes of administration have recently been explored (18–20). The practice of early cord-clamping and cutting has recently been de-emphasized (21), and uterine/fundal massage is now recommended to ensure contraction of the uterus after the delivery of the placenta. These components have been delineated in the recent policy statement of the International Confederation of Midwives and the International Federation of Gynecology and Obstetrics which recommends the practice of AMTSL (22). These three components are clinical events that can be objectively observed and verified.

Postpartum haemorrhage was defined for the purpose of this study as loss of blood after childbirth in excess of 500 mL (23). The definition of postpartum haemorrhage was further restricted to haemorrhage from uterine atony, including atony secondary to retained placenta, which is the most prominent leading cause (with estimates of 80% or greater) of this complication (2). Haemorrhage from cervical or vaginal lacerations was not addressed as this is not affected by AMTSL. Clinical estimates of postpartum haemorrhage typically depend on the judgment of the practitioner with no attempt to verify actual loss of blood through approaches, such as collecting and measuring fluids (24). Visual estimation of blood loss is recognized as inaccurate, especially at the higher limits of measured blood loss (25). Nevertheless, this is the standard and pragmatic approach which is typically combined with a clinical judgment about the health status of women (26, 27) in making a final assessment about further treatment. Treatment as determined by the provider ultimately leads to costs that AMTSL is expected to minimize.

### Estimating probabilities

Reliable estimates of country-specific probabilities for certain study-relevant variables were not readily accessible. Both facility-based statistics and national vital registration records are considered incomplete in both the study countries. Therefore, probability estimates used for calculations of the net benefit of AMTSL came from several sources, including published literature, informed clinicians, and the consensus opinion of expert panels. JHPIEGO staff selected the panel members based on their current academic, administrative or clinical affiliations as likely to be cognizant of contemporary epidemiologic or obstetrical clinical practice issues and patterns. The panel members selected the probabilities of specific events that, in their judgment, best reflected the actual situation in their country.

Estimates of medical probabilities that were required to answer the study question included the likelihood of occurrence of postpartum haemorrhage with EMTSL, postpartum haemorrhage with AMTSL, and mortality, and the probability of each of the expected combinations of clinical management strategies of postpartum haemorrhage. These strategies included: (a) uterotonic (UT) only; (b) uterotonic plus manual removal of the placenta (MRP); (c) uterotonic plus bimanual compression of the uterus (BMC); (d) uterotonic plus MRP and bimanual compression. The probability of transfusion in combination with any of the management strategies was also estimated.

Rates of maternal mortality and postpartum haemorrhage were obtained from publicly-available resources (3, 6, 28, 29). The incidence of postpartum haemorrhage in three randomized clinical trials that compared AMTSL with EMTSL ranged from 5.8% to 6.8% (active) and from 11% to 17.9% (physiologic) when postpartum haemorrhage was defined as loss of 500 mL blood with lower incidence when the volume of blood loss was set at higher level (2, 7–9). We used a 5% rate of postpartum haemorrhage with AMTSL and a 10% rate with EMTSL in this study. These figures were selected based on the recommendation of the expert panels. Country-specific estimates were acknowledged by panel members to be lower-bound, conservative estimates of the actual rates in each country, based on incomplete statistical data, and are, therefore, neither precise nor reliable. If cost-savings could be demonstrated using these lower-bound estimates, cost-savings would be even more certain if the true differences in the rates of postpartum haemorrhage with and without AMSTL were within the higher ranges cited above.

The panel members provided probability estimates for various combinations of management strategies of postpartum haemorrhage as practised in their respective countries. As clinical management strategies for the control of haemorrhage depend, at least in part, on the skill of practitioner and resources at hand (30, 31), the choice of management strategies varies. However, the primary and most often effective clinical intervention is the use of an uterotonic agent. Oxytocin is most commonly used. Experts indicated the relative use of oxytocin and alternative uterotonic agents. Secondary lifesaving interventions to manage postpartum haemorrhage include MRP and bimanual compression of the uterus (32). MRP and bimanual compression can be used individually or together and are virtually always combined with the use of a uterotonic. Although other medical and surgical interventions are available (33–37), their use was so rare in Guatemala and Zambia that they were excluded from the analysis. Experts were also asked about the probability of transfusion.

### Estimating costs

Recurrent costs addressed in these analyses included supplies, resources, and personnel used for providing AMTSL and EMTSL and for managing postpartum haemorrhage. In general, cost estimates were readily available and came from national cost lists and purchasing officers. Estimates of the amounts of supplies used came from two sources. Research personnel observed the third stage of labour in both the countries to determine the supplies used for AMTSL and EMTSL. Experts provided estimates of supplies used during the clinical management of postpartum haemorrhage.

The facility perspective on costs was used in the analysis. The facility's unit costs for materials were determined from purchasing officers and standard country-specific price lists. Patients had to buy their own supplies, or the supplies were donated rather than purchased by the facilities in some cases in both the countries. Every attempt was made to document these practices so that estimates of actual facility costs could be made.

The compensation for services of doctors and nurses and facility costs relating to care of patients were more difficult to document. These were estimated based on the usual staffing patterns, average rates of compensation for several categories of care-givers, average length of stay of the patient for uncomplicated labour and delivery and/or the need for transfusion.

Costs of supplies and personnel for the surgical management of postpartum haemorrhage or its complications were not estimated. Based on available use statistics and on the expressed opinions of informed experts, it was determined that these surgical modalities were very rarely employed.

All prices were translated into US dollars using the prevailing bank rates (August 2004). Information obtained from the expert panel and from literature on probabilities of events and probabilities of clinical management strategies and information on the average number of supplies used in directly-observed clinical events (described below) was combined with the average cost per unit of supplies obtained from purchasing officers and price lists. The result was the average cost per clinical event.

The approaches used for deriving country-specific estimates of the study variables are summarized in Table 1. The probabilities used in the study are presented in Table 2. Certain assumptions were made as the basis for several analytical calculations. These assumptions are documented in the context of discussion of relevant findings.

**Table 1.** Sources of data

Type of data	Data source	Method
Probability of events		
Estimates of probabilities of major perinatal outcomes (PPH, maternal mortality)	Published literature	Internet and library resources
	Verified by expert panel	Group interview
Estimates of probabilities of use of various options for clinical management of PPH	Expert panel*	Group interview
	Informed clinicians	Published literature
	Personal interview	Internet and library resources
Costs of events		
Type and amount of supplies used in AMTSL	Practising clinicians	Direct observation
Type and amount of supplies used in EMTSL	Practising clinicians	Direct observation
Type and amount of supplies used in clinical management of PPH	Expert panel	Group interview
Cost of supplies (personnel and materials)	Informed sources (Personnel in charge of various units, e.g. Human Resources, Laboratory, Purchasing, Blood Bank)	Personal interview
		Published cost lists (e.g. purchase orders, pharmacy price lists)

*Members of the expert panel in Zambia: 2 obstetrician/gynecologists, 3 midwives, 1 physician (other specialty), 1 administrator of a birthing facility, and 1 pharmacy representative

Members of the expert panel in Guatemala: 2 MCH training coordinators, 2 representatives from Ministry of Health, and 5 obstetrician/gynecologists

AMTSL=Active management of the third stage of labour;

EMTSL=Expectant management of the third stage of labour;

PPH=Postpartum haemorrhage

**Table 2.** Probabilities of events

Management and complications of PPH	Guatemala	Zambia
Postpartum haemorrhage		
With AMTSL	0.05	0.05
With EMTSL	0.10	0.10
PPH management strategies		
UT only	0.81	0.39
UT and MRP	0.12	0.22
UT, MRP, and BMC	0.07	0.25
UT and BMC	0.00	0.14
All management strategies	1.00	1.00
Complications (independent probabilities)		
Transfusion	0.10	0.46
Death	0.02	0.09

AMTSL=Active management of the third stage of labour;

BMC=Bimanual compression;

EMTSL=Expectant management of the third stage of labour;

MRP=Manual removal of the placenta;

PPH=Postpartum haemorrhage;

UT=Uterotonic

### Study design

A two-group study designed to measure the costs associated with AMTSL and EMTSL was implemented concurrently in the two study countries. Hospital facilities in both urban and rural settings were selected to reduce any bias that might be introduced by the relative advantages of urban settings. These facilities were also selected to represent various levels of emergency obstetric-care capacity available in the country.

AMTSL has already been demonstrated to be of benefit, and the practice has been recommended for adoption in both the countries (22). Therefore, neither practitioners nor pregnant women were randomized to an intervention group. Instead, actual clinical practices were observed and described. Women were asked for their consent for observation and documentation of the events of their labour, delivery, and postpartum experiences. Verbal consent for this observation was documented on study forms. No personal identifying information was recorded on any study form. Providers (doctor, midwife, nurse, auxiliary nurse) were assured that the choices that they made during clinical management were not the subject of this study and that the quality of their practice was not under review. The ministries of health in both the countries approved the design and implementation of the study in their respective jurisdiction.

Direct observation of births by the study consultants and research assistants allowed for the documentation of types, quantities, and costs of supplies used from the time of birth through a 30-minute observation period following the delivery of the placenta. Management strategies of postpartum haemorrhage or interventions that occurred following this 30-minute observation period were not directly observed, but, rather, were estimated in the analysis based on the probabilities of occurrence developed for the study. In Guatemala, 30 actively-managed and 30 expectantly-managed cases were observed in four sites during May 2004. In Zambia, 23 actively-managed and 15 expectantly-managed cases were observed across six sites from February to April 2004.

The study sites in Guatemala included government hospitals that were each capable of providing emergency obstetric care (EOC). The hospitals were located at distances of 28–218 km from the capital Guatemala City. The number of births per facility ranged from 200 to 500 a month. Clinical providers of care included physicians, nurses, and auxiliary nurses.

The study sites in Zambia included the University Teaching Hospital located in the capital city (Lusaka), three district health centres in the Lusaka community, and two hospitals and one health centre located 150–350 km away from Lusaka. The number of births per facility ranged from 28 to 750 a month. Physicians and registered or enrolled midwives were the clinical providers of care. Each of the hospitals was capable of providing EOC. Patients of the health centres would be referred to these same hospitals, if in need of EOC services.

### Study instruments

A supply checklist was developed and revised through reviews by international medical experts, several in-country clinical observations, and interviews with key-informants in both the countries. The final checklist reflected the usual and customary practices in the country sites. It also reflected the availability of material resources and, therefore, it contained items commonly used as substitutes for items that were out-of-stock or otherwise not available. The country-specific supply checklists differed only by inclusion or exclusion of certain supplies as appropriate for the country.

### Data analysis

#### Cost-benefit analysis

**Fig. 1.**
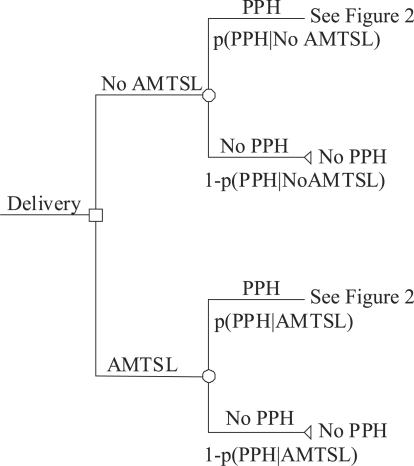
Difference in probability of PPH

**Fig. 2.**
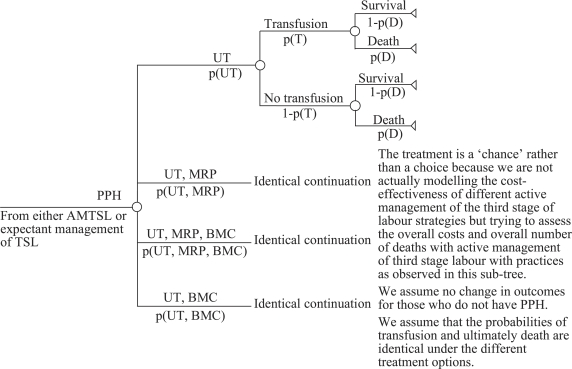
Management and complications

Cost-benefit analysis was made to compare the extra costs of administering AMTSL to a hypothetical population of 100,000 births with expected cost-savings that result from fewer cases of postpartum haemorrhage and associated complications. Figure 1 reflects that the incidence of postpartum haemorrhage depends on the type of management of the third stage of labour. Figure 2 shows the events that occurred after postpartum haemorrhage which contributed to the calculation of the average cost per case of postpartum haemorrhage. The analytical approach used in this analysis assumed the independence of clinical events in the management of postpartum haemorrhage. Providers may choose one or more in various combinations. The choices for clinical management of complications are not influenced by the third stage management approach that was used.

AMTSL=Active management of the third stage of labour;
PPH=Postpartum haemorrhage

AMTSL=Active management of the third stage of labour;
BMC=Bimanual compression;
MRP=Manual removal of the placenta;
PPH=Postpartum haemorrhage;
TSL=Third stage of labour;
UT=Uterotonic

The ‘base case’ analysis used the parameters derived from the literature, expert panel, and observation. (The actual calculations for Guatemala are provided in the Appendix). All savings occur because of decreased risk of postpartum haemorrhage. The costs of postpartum maternal care will be lower for the AMTSL group because there are fewer cases of postpartum haemorrhage. The net costs of AMTSL will be negative (i.e. it will be cost-saving) if the savings are sufficiently large to offset the costs of providing AMTSL to the population. The results may vary between Guatemala and Zambia because of different prices, different probabilities of clinical management strategies of postpartum haemorrhage, and different probabilities of transfusion and death (38). While we did not assign a cost of death from the facility perspective, the decreased number of deaths is useful information for policy-makers.

#### Sensitivity analyses

Three additional sets of analyses were performed to assess the robustness of findings using base case parameters. In the first and primary sensitivity analysis, we varied the probability of different clinical management strategies of postpartum haemorrhage. All 286 combinations of probabilities of four strategies adding up to 100%, with each being a multiple of 10%, were used in sensitivity analysis. This variation was allowed to account for the fact that a slightly different, but potentially confounding, question was asked of respondents in the two countries. Results robust to this set of analyses would indicate that assumptions made about management strategies of postpartum haemorrhage are not driving the result as long as postpartum haemorrhage is managed the same after either AMTSL or EMTSL. In a second sensitivity analysis for Zambia, results were re-calculated using lower rates of transfusion and death, i.e. the mean across the two countries (39).

We kept the cost calculations completely separate in the two countries, recognizing that, between Zambia and Guatemala, the available resources, the medical practices, and both absolute and relative costs are different. Other cost studies reported for multinational clinical trials have combined subjects and used an average cost basis; however, in these cases, participating countries were much more similar on these parameters.

Finally, threshold analyses were conducted for each country. These calculations generated information about the minimum number of percentage points that the probability of postpartum haemorrhage would need to decrease to achieve a positive net benefit from the introduction of AMSTL as a clinical policy and practice.

## RESULTS

Table 3 depicts the base case results for both the countries under theoretical conditions where AMSTL is or is not used. The results for Guatemala demonstrate a net cost-saving of US$ 18,000 and 100 maternal deaths averted if AMTSL were used in 100,000 births. The results for Zambia demonstrate a net cost-saving of over US$ 145,000 and 467 maternal deaths averted when AMTSL is practised in 100,000 births. Both the sets of results indicate that AMTSL is cost-saving at the facility level.

**Table 3.** Cases and costs as a function of AMTSL

Outcome	Zambia		Guatemala	
	With AMTSL	Without AMTSL	With AMTSL	Without AMTSL
	No. of cases			
No PPH	95,000	90,000	95,000	90000
UT, no transfusion, survival	941	1,883	3,605	7,209
UT, no transfusion, death	97	194	74	147
UT, transfusion, survival	815	1,631	381	762
UT, transfusion, death	84	168	8	16
UT/MRP, no transfusion, survival	526	1,053	517	1,034
UT/MRP, no transfusion, death	54	108	11	21
UT/MRP, transfusion, survival	456	912	55	109
UT/MRP, transfusion, death	47	94	1	2
UT/MRP/BMC, no transfusion, survival	617	1,235	310	620
UT/MRP/BMC, no transfusion, death	64	127	6	13
UT/MRP/BMC, transfusion, survival	535	1,070	33	66
UT/MRP/BMC, transfusion, death	55	110	1	1
UT/BMC, no transfusion, survival	344	688	0	0
UT/BMC, no transfusion, death	35	71	0	0
UT/BMC, transfusion, survival	298	596	0	0
UT/BMC, transfusion, death	31	61	0	0
	Unit cost (US$)			
AMTSL	0.52*		0.56*	
UT	3.89		3.86	
MRP	6.57		8.75	
BMC	0.24		0.22	
Transfusion	69.54		97.12	
	Cost for all cases (US$)			
Cost: third stage of labour (without complications)	147,270	95,718	82,755	26,843
Cost: management of complications	196,767	393,535	73,953	147,906
Total cost	344,073	489,253	156,708	174,749
Maternal deaths (number)	467	933	100	200
Net cost	-145,216		-18,041	
Deaths averted (number)	467		100	

*Added personnel costs

AMTSL=Active management of the third stage of labour;

BMC=Bimanual compression;

MRP=Manual removal of the placenta;

PPH=Postpartum haemorrhage;

UT=Uterotonic

The results in Guatemala were robust. The non-negative net benefit did not depend on the proportion of postpartum haemorrhage cases managed with each modality. Holding the probability of postpartum haemorrhage fixed at 10% with expectant management, the threshold analysis indicated that probability of postpartum haemorrhage could be no higher than 6.2% (nearly 80% of the base case effect) with AMTSL to yield cost-savings for the facility.

The findings in Zambia are similarly robust. Again, the non-negative results were invariant to the distribution of management strategies. Re-calculation using lower risks of transfusion and death (the two-country average rather than Zambia's own rate) did not change the cost-saving result. Finally, the threshold analysis was more favourable. AMTSL would result in cost-savings with a probability of postpartum haemorrhage among AMTSL cases as high as 8.7% (less than 40% of the base case effect) rather than 5% used in our analysis.

To summarize, the effectiveness of AMTSL needs to be at least 40% of the base case in Zambia, and this is well under the estimate of 80% of cause-specific (uterine atony) postpartum haemorrhage. In Guatemala, the effect needs to be 80% of the base case for AMTSL to be economically favourable, and this is the same as the estimate of 80% cause-specific postpartum haemorrhage.

## DISCUSSION

The recommendation of the International Confederation of Midwives and the International Federation of Gynecology and Obstetrics (ICM/FIGO) for AMTSL to prevent postpartum haemorrhage is supported by an economic analysis from the facility perspective in a Latin American and sub-Saharan African nation. The results are quite robust. Logic suggests that the general-ly-recommended societal perspective would provide an even stronger economic argument. Given that the deaths prevented are among relatively young women, the number of life-years saved or disability-adjusted life years averted would be substantial. Maternal death may also affect both quality and years of life experienced by children.

This analysis was based on the best information available consistent with the realities of the context of the countries in which we worked. When we made assumptions and analytical decisions we chose to err on the side of less cost-savings. For example, we excluded rarely-used surgery from our analysis, although inclusion would increase the cost-savings from avoiding surgical management of postpartum haemorrhage. We also used a relatively low estimate of the incidence of postpartum haemorrhage with EMTSL, although one study demonstrated a rate as high as 17.9% (7). Greater precision of the incidence estimate of postpartum haemorrhage would increase the precision of the cost-saving results. Nevertheless, the threshold analyses demonstrated that less than a five-percentage point decrease in incidence of PPH with routine practice of AMTSL is necessary to obtain positive net benefits. This finding suggests that policy need not wait for more precise or more valid measures of loss of blood and postpartum haemorrhage.

Similarly, while more precise and valid estimates of the probability of different management strategies of postpartum haemorrhage would also be useful, the sensitivity analyses demonstrate that policy need not wait for these results either. Finally, unless there is a substantial decrease in the probability of transfusion, the expected costs saved from avoided postpartum haemorrhage will continue to be larger than the costs of providing AMTSL to a population.

Training costs were not included in the computations. The techniques of AMTSL are not necessarily new skills for practitioners. AMSTL requires only that clinical practitioners amend the timing of common clinical practices, or introduce slight variations into their manner of practice (e.g. controlled cord traction). It was the case in the two countries in which we worked, that AMSTL was being introduced into pre-service education, and in-service education programming was, in large part, being conducted by NGOs. In the case where either or both of these assumptions is/are not valid, and when in-service education would need to be considered as a cost to the facility, these costs would need to be included in the cost-benefit calculation. However, here training expenses are not anticipated to be a major burden of cost for facility.

Costs relating to the theoretical complications of AMTSL were also not considered. Improper performance of the clinical techniques that comprise the practice of AMTSL can result in the need to implement one or more of the strategies for the management of postpartum haemorrhage. The meta-analyses conducted by Prendiville and Elbourne (10) acknowledge that there is insufficient information about the incidence of complications and side-effects of AMTSL, and no subsequent studies have been reported that enlighten the discussion. Our analysis proceeded on the assumption that the probabilities of these events are reflected in the estimates used in our computations which were based on both reported literature and expert opinion.

The imprecision inherent in the methods that were necessary to generate estimates of the probabilities of clinical events (including various management strategies of postpartum haemorrhage) is the primary limitation to this study. However, various approaches were used for generating these estimates, and sensitivity analyses were used for exploring the robustness of study results against the values of parameters and assumptions. A second limitation is the fact that the study sites were primarily in-hospital facilities. Larger facilities may experience the advantage of certain economies of scale relating to education of provider, materials, and supplies necessary for the implementation of AMTSL. These costs may be higher in other healthcare settings that have greater challenges to access and supply.

Recent work shows significant within-country and international variation in the use of AMTSL despite the apparent economic incentives for adopting it (30, 31). This study adds to the evidence base that can be used for supporting the implementation of AMTSL globally. The study is particularly informative because the study methods were designed to reflect the specific realities and cultural context of the two countries. Evidence alone will not, however, necessarily lead to change (40). The adoption of AMTSL can be facilitated by emerging technology and interest of provider (41, 42). Emerging technologies that can be used by first-line personnel in low-resource settings (43) include single-dose, pre-filled, oxytocin injection devices (44) and rectal administration of misoprostol (which is stable at room temperature) as an alternative uterotonic agent (45).

Finally, a recent study in Zambia included interviews with 140 health professionals and administrators (46). The authors, based on the strong enthusiasm expressed for the principles of quality assurance in that study, recommended that such programmes include broad communication of, and uniform adherence to, common standards of clinical practice, including AMTSL. Other studies support the cost-effectiveness of training providers to implement new practices (47, 48).

The findings of the present study indicate that the proven clinical benefit of AMTSL is also associated with a distinct financial benefit to health facilities. We believe that these findings, although drawn from very cautious cost estimations, assumptions, and procedures, offer a compelling argument in support of the introduction of AMTSL as a clinical practice guideline, with both client and facility benefits as an outcome.

## ACKNOWLEDGEMENTS

This publication was made possible through support provided by the Maternal and Child Health Division, Office of Health, Infectious Diseases and Nutrition, Bureau for Global Health, U.S. Agency for International Development (USAID), under the terms of Award No. HRN-A-00-98-00043-00 for the Maternal and Neonatal Health Program. The opinions expressed herein are those of the authors and do not necessarily reflect those of the USAID. The authors wish to acknowledge the research and consultant staff and members of the expert panels in Guatemala and Zambia, who contributed to this research. The authors also thank Oscar Cordon and Rick Hughes, JHPIEGO country office directors of Guatemala and Zambia respectively, and the JHPIEGO MNH Program and its partners. The authors also thank Judith Robb-McCord and Harshad Sanghvi for their support and input throughout this project.

## Appendix. Calculations of costs for Guatemala

**Table:**

Event	Total no. of cases	Probability of event with AMTSL (%)	Probability of management conditional on PPH (%)	Probability of transfusion result (%)	Probability of mortality result (%)	No. of cases	Cost per case of AMTSL (US$)	Cost of AMTSL for cases of event (US$)	Cost per complication	Cost of complications for cases of event (US$)	Total cost for cases of event (US$)
No PPH	100,000	95				95,000	0.83	78,617.26	0.00	0.00	78,617
PPH	100,000	5				5,000					
All rows below are sub-sets of 5,000 PPH cases											
PPH with UT only, no transfusion, and maternal survival	100,000	5	81	90	98	3,605	0.83	2,982.91	1.61	5,800.64	8,784
PPH with UT only, no transfusion, and maternal mortality	100,000	5	81	90	2	74	0.83	60.88	1.61	118.38	179
PPH with UT only, transfusion, and maternal survival	100,000	5	81	10	98	381	0.83	315.15	88.88	33,846.52	34,162
PPH with UT only, transfusion, and maternal mortality	100,000	5	81	10	2	8	0.83	6.43	88.88	690.75	697
PPH with UT and MRP, no transfusion, and maternal survival	100,000	5	12	90	98	517	0.83	427.88	8.23	4,256.43	4,684
PPH with UT and MRP, no transfusion, and maternal mortality	100,000	5	12	90	2	11	0.83	8.73	8.23	86.87	96
PPH with UT and MRP, transfusion, and maternal survival	100,000	5	12	10	98	55	0.83	45.21	95.50	5,216.82	5,262
PPH with UT and MRP, transfusion, and maternal mortality	100,000	5	12	10	2	1	0.83	0.92	95.50	106.47	107
PPH with UT, MRP, and BMC, no transfusion, and maternal survival	100,000	5	7	90	98	310	0.83	256.73	8.45	2,622.10	2,879
PPH with UT, MRP, and BMC, no transfusion, and maternal mortality	100,000	5	7	90	2	6	0.83	5.24	8.45	53.51	59
PPH with UT, MRP, and BMC, transfusion, and maternal survival	100,000	5	7	10	98	33	0.83	27.12	95.72	3,137.30	3,164
PPH with UT, MRP, and BMC, transfusion, and maternal mortality	100,000	5	7	10	2	1	0.83	0.55	95.72	64.03	65
PPH with UT and BMC, no transfusion, and maternal survival	100,000	5	0	90	98	0	0.83	0.00	1.83	0.00	0
PPH with UT and BMC, no transfusion, and maternal mortality	100,000	5	0	90	2	0	0.83	0.00	1.83	0.00	0
PPH with UT and BMC, transfusion, and maternal survival	100,000	5	0	10	98	0	0.83	0.00	89.10	0.00	0
PPH with UT and BMC, transfusion, and maternal mortality	100,000	5	0	10	2	0	0.83	0.00	89.10	0.00	0
Total cost											138,755
Total maternal deaths						100					

All probabilities come from Table 2

All unit costs come from Table 3

No. of cases=Total cases * (Probability of event with AMTSL) * (Probability of management conditional on PPH) * (Probability of transfusion result) * (Probability of mortality result)

Cost of AMTSL for cases of event=no. of events * cost per case of AMTSL

Cost of complications for cases of event=no. of events * cost per complication

Total cost=Cost of AMTSL for cases of event + cost of complications for cases of event

AMTSL=Active management of the third stage of labour;

BMC=Bimanual compression;

MRP=Manual removal of the placenta;

PPH=Postpartum haemorrhage;

UT=Uterotonic
